# Same Day Biopsy and Treatment of Non-Melanoma Skin Cancer in Patients with Field Cancerization: A Retrospective Chart Study

**DOI:** 10.1155/2023/9990046

**Published:** 2023-02-01

**Authors:** Jonathan Miles, Camila Luis-Gronau, Estefania Cruzval-O'Reilly, Aida Lugo-Somolinos, Puneet S. Jolly

**Affiliations:** Department of Dermatology, University of North Carolina, Chapel Hill, USA

## Abstract

**Background:**

Patients with field cancerization will develop numerous superficial non-melanoma skin cancers (NMSCs). Treating patients with field cancerization can be challenging and burdensome due to the numerous non-melanoma skin cancers (NMSCs) they develop and the frequent dermatology visits required for biopsy and treatment.

**Objective:**

The success rate of diagnosing and treating lesions suspicious for NMSCs on the same day is measured, immediately after biopsy.

**Methods:**

We retrospectively reviewed records of patients with same day lesion diagnosis and curettage treatment to determine diagnostic accuracy, treatment failure, and number needed to treat to reduce a follow-up treatment.

**Results:**

A total of 237 lesions underwent same day biopsy and treatment, of which the majority were NMSC (66%) or actinic keratosis (23%). Patients had at least 3 months and a median of 17 months follow-up. A total of 20 lesions either recurred or were deemed to require additional treatment. The number needed to treat (NNT) to prevent one follow-up treatment was 1.3. Limitations: sample size limited ability to determine risk factors for treatment failure.

**Conclusion:**

Simultaneous diagnosis and treatment of superficial NMSCs is a successful way of improving efficiency and patient satisfaction.

## 1. Introduction

Sun damage from excessive sun-exposure causes changes to the skin which leads to formation of keratinocyte neoplasms (KNs) such as precancerous actinic keratoses (AKs) and subsequent non-melanoma skin cancer (NMSC) such as squamous cell carcinoma [1]. While AKs are not thought to be precursor lesions to basal cell carcinoma (BCC), they are a marker of significant sun damage which is also thought to cause BCC. Over time, sun-damaged keratinocytes develop DNA mutations. These cells then give rise to similarly affected “progeny” cells which leads to “actinic field cancerization” in areas of chronic, unprotected sun-exposure such as scalp, face, and sun-exposed trunk and extremities. Once a patient develops field cancerization on a particular anatomic site, they are at significantly increased risk of developing numerous superficial NMSCs within the affected area(s) [2, 3]. Normally, patients come to see dermatologists where concerning areas are biopsied and sent to a dermatopathologist for analysis. If results show NMSC, patients will return for treatment of one or several lesions by curettage or surgical excision [4]. Certain lesions may also be amenable to topical treatment. It is common to biopsy multiple sites, have the patient return for several visits to address these NMSCs, and then have the patient return again within a few months or even weeks to repeat the process [3]. The cycle gives rise to “treatment fatigue” for patients who may actually stop coming for routine screening visits. It also leads to reduced access to other patients who could use these clinic visits. Finally, it results in a significant cost to our health care system. The average number of individuals treated for skin cancer in the United States rose from 3.4 to 4.9 million between 2002–2006 and 2007–2011 while the cost for treating skin cancer rose 126%, from 3.6 to 8.1 billion dollars [5]. Annual spending for skin cancers increased more than for any other cancer [6].

Many dermatologists have recognized these challenges and have anecdotally biopsied and treated suspected NMSCs at the time of the visit to help address these concerns. This same day biopsy and treatment serve to accomplish several things such as (i) increase efficiency: by addressing concerning lesions at the time of presentation, it would eliminate the need for subsequent visit, (ii) improve patient satisfaction and reduce “treatment fatigue, and (iii) reduce cost to our health care system: if treatment is performed at time of the biopsy, providers cannot bill the cost of both the biopsy and the treatment. If the lesion is a skin cancer, the provider can only bill the cost of the treatment and not the biopsy. Conversely, if the lesion is benign, only the biopsy can be billed to insurance. One barrier to utilizing this technique is concern about efficacy of treatment as well as overtreatment of benign lesions. We sought to examine these concerns in addition to other areas of interest including efficacy related to skin cancer type, location, and immune status.

## 2. Methods

### 2.1. Patient Selection and Treatment

This study was approved by the UNC institutional review board. Patients from UNC Dermatology Clinic were included in this chart review if they were found to have lesions concerning for superficial skin cancers such as sBCC, SCCis, and early nodular BCC and were amenable to this treatment approach. Lesions were anesthetized using 1% lidocaine with epinephrine (1 : 100,000), and shave biopsy was performed. Immediately following biopsy, sites were treated with curettage with or without light electrodessication. Patients were subsequently monitored with regular follow-up.

### 2.2. Statistical Analysis

Medical records were reviewed for demographic information, immunosuppression status, history of NMSC, and lesion characteristics, including diagnosis, location, and size. Data were stored in an online secure database, RedCap [7] and analyzed with Stata, version 16 (StataCorp LLC, College Station, TX). Primary outcomes were diagnostic accuracy and treatment failure. Diagnosis was considered accurate if histopathology identified a keratinocytic neoplasm (NMSC or AK). All other lesions were classified as benign. Treatment failure was defined as tumors that either recurred or were deemed to require additional treatment after initial histopathological analysis was performed. Secondary outcomes were predictive relationships between patient and lesion characteristics with treatment failure as assessed by univariate and multivariate logistic regression, correlation between patient and lesion characteristics with treatment failure as assessed by *X*^2^ tests, unpaired *t*-test, or Fisher exact tests and lastly calculating the number needed to treat (NNT) to prevent follow-up treatment.

## 3. Results

From July 2016 to October 2019, 63 patients received same day biopsy and treatment with curettage of 244 lesions and were followed for at least 3 months. Lesion characteristics are summarized in Table 1. Majority of lesions occurred on male patients (81%) at a median age of 73 years. In total, 211 lesions (89%) were pathologically confirmed as KN (Table 2) demonstrating either squamous cell carcinoma in situ (SCCis; *n* = 71), invasive squamous cell carcinoma (iSCC; *n* = 27), basal cell carcinoma (BCC; *n* = 62), or actinic keratosis (AK or hypertrophic AK (HAK); *n* = 57). The 28 benign diagnoses are listed in Table 2.

Median follow-up time was 32 months (interquartile range 20–40.5), during which time 23 lesions (10.6% of 216 KNs) failed treatment, either by recurring (*n* = 6) or requiring additional treatment (*n* = 17) due to incomplete resolution of the initial lesion. Failure occurred in 10 of 28 (36%) iSCC, 8 of 68 (12%) SCCis, and 5 of 64 (8%) BCC. No treatment failures occurred in any of the 56 AK (Table 3). Seven of the ten iSCC failures were in lesions ranging from 10 to 19 mm versus iSCC less than 10 mm (2 of 10). One iSCC was missing size information. There were no iSCC lesions greater than 19 mm in this study. Of the 12 SCCis and BCC lesions treated that were greater than 19 mm, only four SCCis lesions were considered treatment failures.

Univariate analysis of a *priori* patient and lesion characteristics from Table 1 found only location on the face, ears, or neck to be significantly related to failure (OR 15.7, 95% CI: 1.87–132; *p* = 0.011) using lesions on the trunk as the reference category. Age, sex, immunocompromised status, and size category each had no significant relation to failure. Lesion histopathology, a potential mediating variable, was significantly related to treatment failure in a Fisher exact test (*p* ≤ 0.001; Table 3) and in univariate logistic regression with iSCC showing an OR of 6.56 (95% CI: 1.98–21.7; *p* = 0.002) using BCC as the reference category. A multivariate logistic regression model included location, size category, and histological type. In this model, iSCC and histological type both maintained significance with respective ORs of 15.5 (1.40–131; *p* = 0.025) and 7.19 (1.75–29.4; *p* = 0.006). Size category became significant with size greater than 19 mm showing an OR of 6.07 (1.17–31.4; *p* = 0.031) when compared to size less than 10 mm.

Using the data from all lesions in this sample as an estimate for the rates of benign, cancerous, and pre-cancerous lesions in biopsied specimens, we estimate that 89% of biopsies would require follow-up treatment after being identified as either NMSC or AK. Same day treatment reduces the rate of follow-up treatment to 10.6%. This is an absolute risk reduction of 78% and a NNT of 1.3. Excluding actinic keratosis in the calculations gives a more conservative NNT of 2.0 given a baseline follow-up treatment rate of 66% reduced to 14%.

## 4. Conclusion

Efficiency, cost savings, and patient satisfaction are becoming increasingly valued metrics in medicine. In efforts to save patients time, decrease cost, and increase efficiency, we have been treating lesions suspicious for superficial NMSCs with curettage immediately after biopsy. Risk of “overtreatment” of benign lesions and “under treatment” of invasive lesions was reviewed prior to biopsy. When presented with the option of curettage at time of biopsy, we observed that patients preferred this to waiting for results and scheduling a return visit for treatment (although this was not conducted as a formal survey). An important prerequisite for performing this technique is having a high pretest probability in identifying superficial KN. Therefore, we examined success and failure rates as the ability to distinguish superficial NMSC from benign lesions. There is much variability in the literature describing rates of NMSC diagnosis in patients undergoing biopsy concerning for possible skin cancers. Some reports describe approximately 50% rate of diagnosing NMSC [8] to over 85% [9]. We report a 66% rate of NMSC diagnosis (BCC or SCC) increasing to 89% when including AK and HAK.

Another component of measuring success had to do with how well we were able to eliminate the need for further treatment, whether it is cryotherapy, topical therapy, curettage, or excision. By performing curettage immediately after biopsy, the intention was to eliminate the need for additional treatment or visits to confirm clearance if a topical agent was utilized. We found a large reduction in need for further treatment with a NNT to reduce one follow-up treatment of 1.3 to 1.9. In other words, same day biopsy and curettage of 100 lesions would over treat 11 benign lesions while sparing 51 to 78 lesions from needed follow-up treatment.

Curettage with or without electrodessication has been employed by dermatologists since the beginning of the 20^th^ century [10]. There are numerous reports describing success and recurrence rates in treating NMSC. An important study by Silverman et al. from 1991 measured recurrence of BCC treated by ED&C from 1955 to 1982 at NYU [11]. This study shaped many of the modern principles and guidelines regarding the use of curettage with and without electrodessication in dermatology. Higher rates of recurrence were found for BCCs treated on the nose, chin, ear, and periorificial locations while lowest risks of recurrence were found on the trunk, extremities, and neck. Lesions less than 1 cm showed a 9-10% 5-year recurrence (all sites). However, recurrence rates in lower risk areas, regardless of lesion diameter, were only between 2 and 4%. Another important consideration is subtype of BCC. Infiltrative and morpheaform subtypes are not amenable to ED&C, and it is estimated that ∼13% of BCCs show either of these subtypes [12]. Same day biopsy and curettage for BCC in our analysis showed an 8% failure rate as measured by recurrence or need for additional treatment which is consistent with prior reports. There were no infiltrative or morpheaform BCCs biopsied or treated in this analysis.

Many studies have demonstrated curettage with or without electrodessication to be a valuable treatment option for SCCis with estimates of success averaging between 93 and 98% [13]. Most reports exclude SCCis with follicular or adnexal involvement since cure rates are much lower for this subtype. The data presented in our analysis showed an 88% overall success rate in treating SCCis. An additional concern was presence of iSCC found in previously biopsied SCCis discovered during standard excision or Mohs which has been described [14]. While this has been described, there were no such cases discovered in this analysis.

Invasive SCC is typically treated surgically by standard excision or Mohs micrographic surgery. Nonetheless, numerous studies including recent reports have demonstrated excellent success treating lesions with curettage. A recent report showed a 97% success rate using curettage alone to treat iSCC [15]. Success rate in our analysis was 64%. It is important to note that 4 lesions that were not adequately treated by same day biopsy and curettage were treated by Mohs.

While about a third of lesions treated were on immunocompromised patients, almost half of treatment failures came from lesions on these patients. However, in statistical analyses, immunocompromised status did not confer significant increased risk of treatment failure. All of these lesions that failed treatment were either invasive SCC or SCCis. It is well-known that immunosuppressed patients are at increased risk of recurrence as well as distant spread so the decision was made in many of these cases to refer to Mohs [16].

Treatment success was significantly different between lesion locations, demonstrating the highest success rate on the trunk (98%) compared to the face, ear, or neck locations with a success rate of 77%. Unsurprisingly, success rate varied significantly by histology. Same day curettage showed a 92% success rate for BCC (combining nodular and superficial types) while success rates for SCCis and iSCC were 88% and 64%, respectively. Importantly, there was no significant difference between success rates in well-differentiated vs. moderately differentiated SCCs treated at the time of biopsy (55% vs. 56%, data not shown). Additionally, there was no significant difference between success rates in superficial or nodular BCC (95% vs. 90%, data not shown). While success rates did not significantly vary by lesion size, there was a trend towards lower success with larger lesions. However, it is worth noting that success rate for SCCis less than 2 cm was 93% (56/60). Success rate for SCCis lesions measuring greater than 2 cm was only 50%. Success rate for BCCs less than 2 cm was 91% (53/58).

This analysis would have benefitted from more patients and a greater number of tumors. Few treatment failures limit the interpretation of statistical analyses to evaluate potential predictive variables or confounders for treatment failure.

Overall, there was a high success rate in treating lesions suspicious for NMSC immediately following biopsy; the vast majority (91%) of the tumors showed no evidence of recurrence with a minimum of 3 months follow-up. This practice shows a large reduction in need for follow-up appointments at the cost of a few benign lesions undergoing treatment. The decision to same day treatment should be a mutual decision between provider and patient. This analysis adds data to inform that discussion.

We conclude that this approach should be considered for treating clinically diagnosed NMSC lesions on relatively small- and superficial-appearing lesions on immunocompetent patients. Caution should be exercised in treating lesions on the face, larger lesions, or tumors in immunocompromised patients with curettage. Also, this method is not appropriate for lesions that clinically are suspected to have an infiltrative or deeply invasive growth pattern. Providers who want to carry out this technique should always ensure regular follow-up and monitor the lesions routinely. Altogether, our hope is that this study demonstrates the feasibility of treating superficial skin cancers at the time of biopsy to improve patient satisfaction, efficiency, and cost savings to our healthcare system.

## Figures and Tables

**Table 1:** Descriptive characteristics of 244 lesions among 63 patients.

Characteristics	Value^1^
Male	198 (81%)
Age (years)^2^	73 (65–77)
Immunocompromised	92 (38%)
Transplant	73 (79%)
Other	19 (21%)
Prior NMSC count^2^	21 (11–32)
Location	
Face/ears/neck	35 (14%)
Scalp	28 (11%)
Trunk	54 (22%)
Upper extremity	89 (36%)
Lower extremity	38 (16%)
Size (mm)^2^	
<10 mm	106 (43%)
10–19 mm	104 (43%)
>19 mm	22 (9%)
Missing	12 (5%)

LE, lower extremity; NMSC, non-melanoma skin cancer; UE, upper extremity. ^1^All measurements listed as *n* (%) unless otherwise marked. ^2^Reported as median (*Q*1–*Q*3).

**Table 2:** Histopathological diagnoses of 244 lesions among 63 patients.

Type							
SCCis	71 (29%)			SCC	95 (40%)	KN	211 (89%)
iSCC (KA type)	8 (3%)	iSCC	27 (11%)
iSCC (well diff)	11 (4%)						
iSCC (mod diff)	8 (3%)						
sBCC	22 (9%)			BCC	62 (26%)
nBCC	41 (17%)						
AK	32 (13%)			AK	57 (23%)
HAK	25 (10%)						
Benign^1^	28 (11%)				

AK, actinic keratosis; BCC, basal cell carcinoma; HAK, hypertrophic actinic keratosis; iSCC, invasive squamous cell carcinoma; SCCis, squamous cell carcinoma in situ, ^1^Benign includes verruca (*n* = 7), seborrheic keratosis (*n* = 5), scar (*n* = 3), and other (*n* = 13).

**Table 3:** Factors associated with treatment failure among 244 lesions.

Variables	Treatment success (*n* = 221; 91%)	Treatment failure (*n* = 23; 9%)	*p* value
Male	178 (81%)	20 (87%)	0.454 ^ 1
Age (years)^1^	73 (65–77)	74 (68–78)	0.331 ^ 2
Immunocompromised	82 (37%)	10 (43%)	0.550^2
Transplant	64 (76%)	9 (90%)	0.642 ^ 3
Other	18 (22%)	1 (10%)	
Prior NMSC count^2^	21 (11–32)	22 (11–30)	0.682 ^ 2
Location^5^			
Face/ears/neck	27 (77%)	8 (23%)	**0.024 ^ 3**
Scalp	25 (96%)	3 (4%)	
Trunk	53 (98%)	1 (2%)	
UE	82 (92%)	7 (8%)	
LE	34 (89%)	4 (11%)	
Size^5^			
<10 mm	97 (92%)	9 (8%)	0.361 ^ 3
10–19 mm	95 (91%)	9 (9%)	
>19 mm	18 (82%)	4 (18%)	
Missing	11 (92%)	1 (8%)	
Histological type^5^			
Benign^4^	N/A	N/A	**<0.001 ^ 3**
Invasive SCC	18 (64%)	10 (36%)	
SCC in situ	60 (88%)	8 (12%)	
BCC	59 (92%)	5 (8%)	
AK	56 (100%)	0 (0%)	

BCC, basal cell carcinoma; LE, lower extremity; SCC, squamous cell carcinoma; UE, upper extremity. ^1^Chi squared test. ^2^*t*-test. ^3^Fisher exact test. ^4^Benign not counted in “treatment success.” ^5^Percentages reported are across the row.

## Data Availability

The data used to support the findings of this study are available from the corresponding author upon request.
